# Influence of Basalt Fiber Morphology on the Properties of Asphalt Binders and Mixtures

**DOI:** 10.3390/ma17215358

**Published:** 2024-11-01

**Authors:** Chenhao Cai, Keke Lou, Fuxin Qian, Peng Xiao

**Affiliations:** 1 College of Civil Science and Engineering, Yangzhou University, Yangzhou 225127, China; 221404602@stu.yzu.edu.cn (C.C.); pengxiao@yzu.edu.cn (P.X.); 2 Administration of Urban Facilities, Yangzhou 225003, China; 18936272588@163.com

**Keywords:** basalt fiber, asphalt binder, asphalt mixture, rheological behavior, pavement performance

## Abstract

Basalt fiber (BF) has been proven to be an effective additive for improving the properties of asphalt mixtures. However, the influence of basalt fiber morphology on the properties of asphalt binders and mixtures remains inadequately explored. In this study, chopped basalt fiber (CBF) and flocculent basalt fiber (FBF) were selected to make samples for testing the influence of the two types of basalt fibers on asphalt materials. Fluorescence microscopy was used to obtain the dispersion of fiber in asphalt binders. Then, a temperature sweep test and a multiple stress creep recovery (MSCR) test were carried out to appraise the rheological characteristics of the binder. Moreover, the performance of the fiber-reinforced asphalt mixture was evaluated by a wheel tracking test, a uniaxial penetration test, an indirect tensile asphalt cracking test (IDEAL-CT), a low-temperature bending test, a water-immersion stability test, and a freeze–thaw splitting test. The results indicate that the rheological behavior of asphalt binders could be enhanced by both types of fibers. Notably, FBFs exhibit a larger contact area with asphalt mortar compared to CBFs, resulting in improved resistance to deformation under identical shear conditions. Meanwhile, the performance of the asphalt mixture underwent different levels of enhancement with the incorporation of two morphologies of basalt fiber. Specifically, as for the road property indices with FBFs, the enhancement extent of DS in the wheel tracking test, that of R_T_ in the uniaxial penetration test, that of the CT_index_ in the IDEAL-CT test, and that of ε_B_ in the low-temperature trabecular bending test was 3.1%, 6.8%, 15.1%, and 6.5%, respectively, when compared to the CBF-reinforced mixtures. Compared with CBFs, FBFs significantly enhanced the elasticity and deformation recovery ability of asphalt mixtures, demonstrating greater resistance to high-temperature deformation and a more pronounced effect in delaying the onset of middle- and low-temperature cracking. Additionally, the volume of the air void for asphalt mixtures containing FBFs was lower than that containing CBFs, thereby reducing the likelihood of water damage due to excessive voids. Consequently, the moisture susceptibility enhancement of CBFs to asphalt mixture was not obvious, while FBFs could improve moisture susceptibility by more than 20%. Overall, the impact of basalt fibers with different morphologies on the properties of asphalt pavement materials varies significantly, and the research results may provide reference values for the choice of engineering fibers.

## 1. Introduction

**Figure 1:**
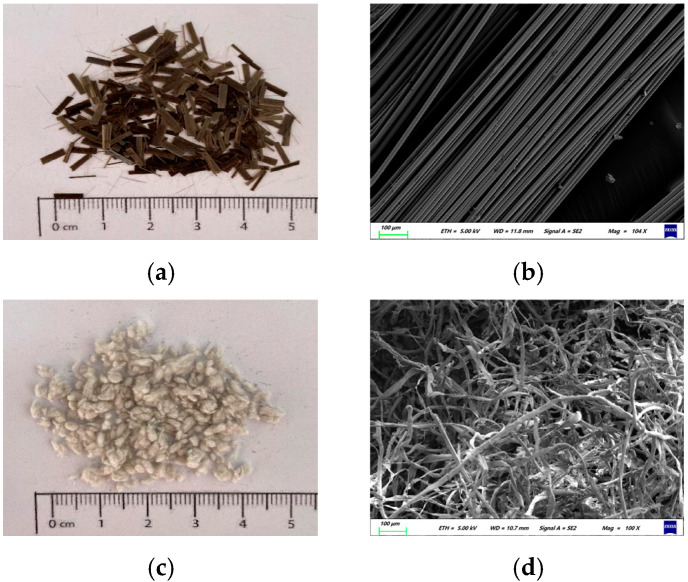
Basalt fibers: (**a**) CBFs; (**b**) microstructure of CBFs; (**c**) FBFs; (**d**) microstructure of FBFs.

Continuous basalt fiber, a kind of amorphous inorganic nonmetallic fiber, is made from basalt ore as the raw material by melting at 1450~1500 °C and rapid drawing of the platinum–rhodium alloy leakage plate [1,2]. For different purposes, continuous basalt fiber is reprocessed into different products to meet diverse needs [3,4]. There are two main types of basalt fibers: chopped basalt fiber (CBF) and flocculent basalt fiber (FBF) [5,6,7,8,9,10]. CBFs, as shown in Figure 1a,b, are made from continuous basalt fibers by cutting them to a certain length and dimension, whereas FBFs (shown in Figure 1c,d) are produced from continuous basalt fibers by dispersion, shearing, and granulation after the centrifugal or blowing-out process, leading them to have a rougher surface and a smaller diameter.

As a kind of durable pavement material with excellent mechanical properties and low economic cost, basalt-fiber-reinforced asphalt mixture has been widely used [11]. At present, scholars mainly focus on three aspects of CBF on the performance and damage characteristics of asphalt binders or asphalt mixtures: content, length, and diameter [12,13]. The investigation of Lou [14] on the macro-performance of ultra-thin wearing course with different fiber types proved that the basalt fiber with an optimum content of 0.2~0.3% exhibited excellent performances at high or low temperatures. Sun [15] pointed out that when the content of basalt fiber in asphalt mixture reaches 0.4% by weight, the ultimate tensile stress of asphalt mixture reaches a peak value. Zhang [16] added different types of fibers with contents of 0.15%, 0.3%, and 0.45% into the open-graded friction course (OGFC) asphalt mixture, and focused on evaluating the drainage performance, rutting resistance, stiffness, and fatigue life of the mixture. Some scholars have evaluated the adaptability of fiber length to hot-mixed asphalt mixtures with different nominal maximum particle sizes of aggregates; then, an optimum length for CBFs for asphalt mixtures with different gradations and maximum nominal grain sizes was proposed [17,18,19,20]. In addition, relevant studies have shown that [17,21,22] a stone-matrix asphalt (SMA) mixture has a skeleton structure with large spacing between coarse aggregates, so longer basalt fibers can play a better bonding role in the mixture. Meanwhile, research on the influence of basalt fiber diameter on the performance of asphalt mixtures has been performed recently. Wu et al. [23] found that the bond strength between the three kinds of short-cut basalt fiber with a diameter in the range of 13–16 μm and asphalt is greater than that of polyester fiber or polyacrylonitrile fiber under the condition of 25 °C. Pei [13] selected basalt fibers with three different diameters to conduct comparative analyses on the crack resistance of asphalt mixture and evaluated the crack inhibition effect of fiber diameters on asphalt mixtures.

However, FBFs have attained the attention of researchers slightly late, and research into the application of FBFs in pavement engineering has gradually emerged only recently. In Shi’s and Kou’s studies, chopped basalt fiber, flocculated basalt fiber, polyester fiber, and lignin fiber were adopted to compare and analyze the reinforcement effects on the asphalt binder [24,25]. The results revealed that flocculated basalt fiber could heighten the high-/low-temperature properties of asphalt mastic, which showed better improvement in elasticity and deformation recovery. Wu and Gu [23,26] compared and measured the moisture absorption, pH value, and asphalt adsorption capacity of three kinds of CBFs, coated with different treating compounds, and one kind of FBF; then, they discussed the rheological properties and micro-morphologies of the asphalt binders. Research on FBFs is limited at present, and most studies focus on their effects on asphalt mortar. Furthermore, the properties of the two fibers are very different, and there is a lack of research into comparative analyses between FBFs and CBFs in relation to their influence on the properties of asphalt mixtures.

Therefore, the preliminary evaluation and comparison of an asphalt–binder mixture blended with basalt fibers were conducted to explore the effect of different morphologies of basalt fibers on the overall performance of asphalt pavement materials in this study. The temperature sweep and MSCR tests were carried out on fiber–asphalt binder samples to evaluate the rheological properties. The original asphalt mixture, the CBF-reinforced asphalt mixture and the FBF-reinforced asphalt mixture were prepared to test the high-temperature, medium-temperature and low-temperature performance and moisture susceptibility via a wheel tracking test, a uniaxial penetration test, an IDEAL-CT test, a low-temperature trabecular bending test, a water immersion stability test as well as a freeze–thaw splitting test. This study analyzes the interaction between basalt fibers and asphalt, elucidating the enhancing mechanisms of basalt fibers in asphalt materials. Furthermore, the impact of basalt fiber on the performance of asphalt pavement is examined. The findings of this research provide valuable insights for road management professionals, enabling them to select basalt-fiber-reinforced materials that exhibit superior performance and are more appropriate for specific engineering scenarios. Ultimately, this can lead to an extension in pavement service life, a reduction in maintenance frequency and costs and an enhancement in the overall road service quality.

## 2. Materials and Test Methods

### 2.1. Raw Materials and Sample Preparation

#### 2.1.1. Fiber

CBFs and FBFs, produced by Jiangsu Tianlong Basalt Continuous Fiber Co., Ltd., Yangzhou, China, were adopted. CBFs are golden brown, straight and free of impurities, while FBFs are dark gray cotton-like particles with different sizes. The technical specifications of the two fibers are summarized in Table 1.

#### 2.1.2. Asphalt

Styrene-butadiene-styrene block copolymer (SBS)-modified asphalt (PG 76-22) was acquired from Nantong Tongsha Asphalt Technology Co., Ltd. (Nantong, China). The related property indicators were tested in strict accordance with the specifications [27]. The softening point of asphalt was 84 °C and the viscosity at 135 °C was 1.8 Pa∙s, respectively.

#### 2.1.3. Aggregate and Filler

The nominal maximum sieve size of aggregates is 13.2 mm in this experiment, and the filler is the limestone mineral powder, with a moisture content of 0.3% and a proportion of 92.2%. The particle size is less than 0.075 mm. The aggregate performance tests, etc. were carried out according to JTG E42-2005 [28], and all the indices met the requirements of the specification.

#### 2.1.4. Fiber–Asphalt Binder

**Figure 2:**
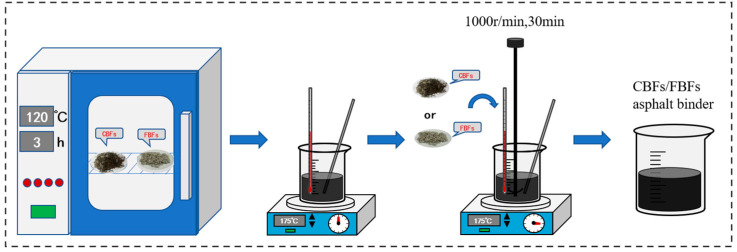
Mixing process of fiber–asphalt binders.

Generally, relevant studies have shown that the weight content of the basalt fiber in asphalt mixture varies from 0.1% to 0.4%. Therefore, the fiber weight content in the SBS-modified asphalt was selected from 1% to 4%, setting 1% as the interval [29,30], and the asphalt without fiber was treated as a control group. Figure 2 illustrates the mixing process of the fiber–asphalt binder samples. The detailed steps are as follows. First, the fibers were placed in an oven at 120 °C for 3 h to avoid the influence of moisture on the test results. Next, the asphalt was placed on a temperature control plate and heated to 175 °C. Then, the fibers were divided into 3 parts and were slowly added to the asphalt in batches. The fibers were then stirred at 1000 RPM for 30 min. The temperature of the fiber–asphalt binder was maintained at 175 °C ± 5 °C throughout the preparation process. In addition, the SBS original asphalt binder would also undergo the above manufacturing process to eliminate the effects of aging.

#### 2.1.5. Gradation Design

**Figure 3:**
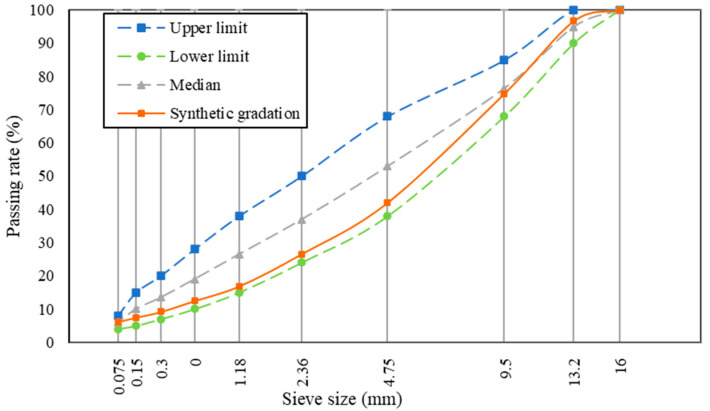
The design grading curve of AC-13.

The AC-13 graded asphalt mixture was designed by the Marshall design method. Generally, 2.36 mm is the key control sieve size, and its passing rate is lower than the median value of the target sieve size range when the mixture gradation is the coarse gradation. Owing to the better skeleton structure and high temperature stability, the coarse gradation of AC-13 was selected in this paper. After several trials, the final design grading curve is reported in Figure 3.

According to the differences between the additives, three types of asphalt mixtures were fabricated, namely neat asphalt mixture, CBF-reinforced asphalt mixture, and FBF-reinforced asphalt mixture, respectively. Based on the engineering practice and the previous research results of the research group [12,19,31], the proposed weight content of basalt fiber in asphalt mixture was selected as 0.3%. Therefore, the 0.3% content of basalt fiber was used to prepare asphalt mixture samples. The optimum asphalt content (OAC) was determined by the Marshall test [27]. The composition materials of different asphalt mixtures and Marshall test results are listed in Table 2. Among them, the volume parameter data of air void (VV), void in mineral aggregate (VMA) and void filled with asphalt (VFA) were kept at similar levels, which could help to better evaluate the influence of other factors on the performance of the asphalt mixtures.

It was found from Table 2 that in the asphalt mixtures, the mixture with FBFs has the largest OAC value, followed by that with CBFs. This is because basalt fiber has a certain oil absorption ability. Under the same weight content of fiber, FBFs have wider spatial distribution and greater total contact area with asphalt.

### 2.2. Test Methods

#### 2.2.1. Asphalt Binder Tests

##### Fluorescence Microscopy

**Figure 4:**
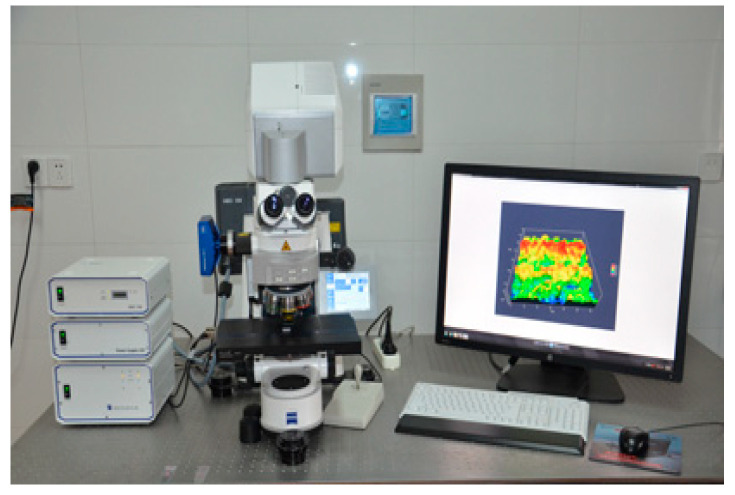
LSM 700 3D measurement laser microscope.

Under certain fluorescent irradiation conditions, the basalt fiber coated with the infiltrator and SBS modifier will show a yellow (bright) color; by contrast, the asphalt will show a black (dark) color. Therefore, the LSM 700 3D measurement laser microscope, as shown in Figure 4, produced by Carl Zeiss in Germany was used to capture the distribution photo of fibers in the asphalt binders. A 405 nm solid-state laser was adopted to acquire the fluorescence microscopic images of fiber–asphalt binders at 100 μm magnification. The steps to prepare the observed samples were as follows:

(1) First, a metal container with a diameter of 5–10 mm and a height of 15–20 mm was placed on a horizontal table;
(2) Second, the different asphalt binders were uniformly poured into metal containers and kept at room temperature for half an hour;
(3) Next, the samples were demoulded and then placed in a refrigerator at −24 °C for 4 hours;
(4) Finally, the frozen samples were taken out and cut immediately, so as to obtain a relatively flat observation surface.

##### The Temperature Sweep Test

According to AASHTO T315 [32], the temperature sweep test was conducted to investigate the impact of fiber morphologies on the rheological properties. The test equipment is the DHR-2 rheometer produced by TA Instrument of Waters Company, New Castle, DE, USA. The test method is suitable for the determination of phase angle (δ), complex shear modulus (G*) and rutting factor (G*/sinδ) of the asphalt binders. The diameter of the sample is 25 mm and the thickness is 1 mm. A sinusoidal vibration load with an angular frequency of 10 rad/s was applied to the testing samples. And the test temperature was from 52 °C to 82 °C, with an increment temperature of 6 °C.

##### Multi-Stress Creep Recovery (MSCR) Test

The test temperature of the MSCR test was set as 64 °C, and the test equipment was the same as the temperature sweep test. According to AASHTO T350 [33], the test samples were loaded 10 times under two stress levels (0.1 kPa and 3.2 kPa). During each loading cycle, the creep loading time is 1 second and the recovering time is 9 seconds. The samples were placed between two parallel stainless steel plates with a diameter of 25 mm, and the gap value was set as 1000 μm. Nonrecoverable creep compliance (J_nr_), elastic recovery rate (R) and relative difference in nonrecoverable creep compliance (J_nr-diff_) were used to analyze the rutting resistance of asphalt binders.

#### 2.2.2. Asphalt Mixture Tests

##### Wheel Tracking Test

The wheel tracking test was conducted in accordance with the JTG E20-2011 specification [27] to assess the high-temperature deformation resistance of asphalt mixtures. The samples with the size of 300 mm × 300 mm × 50 mm were prepared, and the temperature of the instrument chamber was preheated to 60 °C before loading. During the test, the loading speed was set to 42 cycles per minute and the loading stress was set to 0.7 MPa. The dynamic stability (DS) was measured to appraise the high-temperature properties of the samples. Usually, a higher DS value indicates a better resistance of the asphalt mixture against rutting damage.

##### Uniaxial Penetration Test

**Figure 5:**
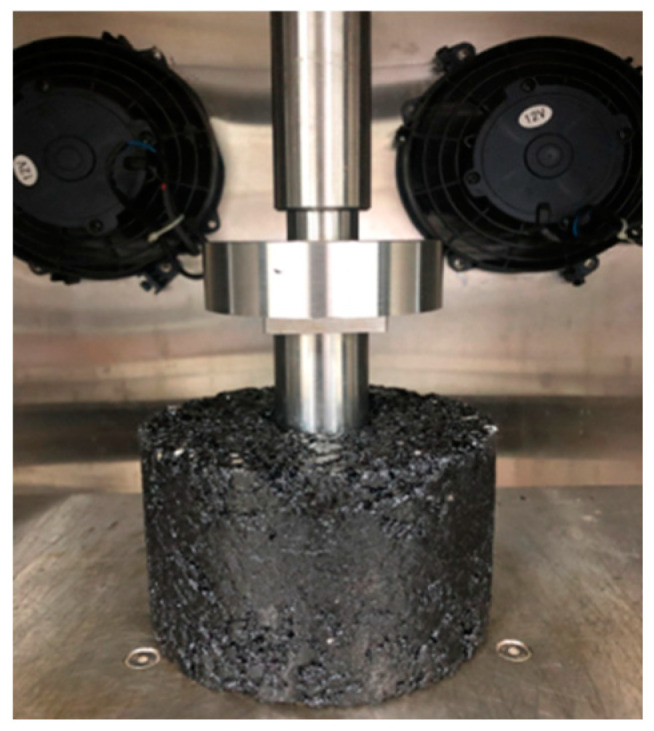
Loading diagram of uniaxial penetration test.

Uniaxial penetration tests can not only measure the shear strength of asphalt mixtures but also reflect the actual stress state of the asphalt materials in the pavement structure [34]. According to the JTG D50-2017 specification [35], cylindrical samples with a diameter of 150 mm and a thickness of 100 mm were prepared by the rotating compactor. The samples were placed into the chamber of the UTM-25 testing machine, and a metal indenter with a diameter of 42 mm and a thickness of 50 mm was placed above the samples. The test loading rate and temperature were set as 1 mm per minute and 60 °C, respectively. The loading diagram of the uniaxial penetration test is shown in Figure 5. The penetration strength (*R_T_*) is calculated in compliance with Equations (1) and (2) [36].

(1) $$\sigma_{P}=\frac{P}{A}$$

(2) $$R_{T}=f_{T}\sigma_{P}$$

where *P* is the maximum load, *N*; *A* is the contact area, mm^2^; *f_T_* is the coefficient of penetration stress with a value of 0.35.

##### Indirect Tensile Asphalt Cracking Test (IDEAL-CT)

**Figure 6:**
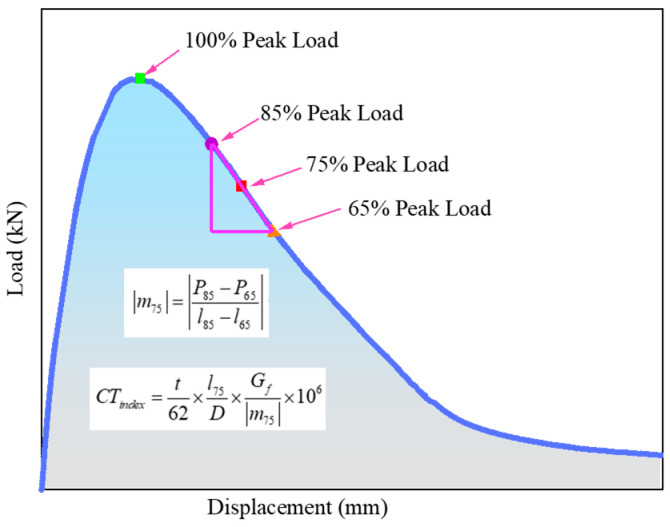
The load–displacement curve of IDEAL-CT test.

The IDEAL-CT test was adopted to test the cracking resistance of asphalt mixtures at medium temperatures according to ASTM D8225-19 [37]. Figure 6 presents the load–displacement curve of the experiment. It can be divided into two stages: the left part before the 100% peak load exists is called the crack initiation stage, while the right part is called the crack propagation stage [38]. Therefore, this test focuses on capturing the load value and the corresponding vertical displacement. CT_index_ is determined by analyzing the load–displacement curve, and the larger CT_index_ value means better anti-cracking ability. In this study, samples with a diameter of 150 mm and a thickness of 62 mm were formed by the rotary compactor. The compression load was applied to the sample at 25 °C under a constant rate of 50 mm/min. It was worth mentioning that the cylindrical asphalt mixture samples were subjected to indirect tension along the diameter plane direction.

##### Low-Temperature Bending Test

According to the JTG E20-2011 specification [27], a low-temperature bending test was also conducted to evaluate the cracking resistance of three asphalt mixtures. The samples with a length of 250 mm, a width of 30 mm and a thickness of 35 mm were placed in a fixed temperature container at −10 °C for 4 h. Then, a point load was applied to the middle span of trabecular samples with a speed of 50 mm/min.

##### Water Immersion Stability Test and Freeze–Thaw Splitting Test

The asphalt mixtures were subjected to a combination of a water immersion stability test and a freeze–thaw splitting test, which could better reflect the anti-moisture damage of asphalt mixtures [39]. Residual stability (MS_0_) was defined by the ratio of the Marshall stability value of the asphalt samples immersed in hot water (60 °C) for 48 h and for 0.5 h. Meanwhile, the freeze–thaw splitting tensile strength ratio (TSR) was the splitting strength ratio of the samples after and before the freeze–thaw process. The two indices were adopted to appraise the moisture susceptibility of samples.

## 3. Experimental Results and Analysis

### 3.1. Properties Evaluation of Fiber-Reinforced Asphalt Binders

#### 3.1.1. Fluorescence Microscopy Test

**Figure 7:**
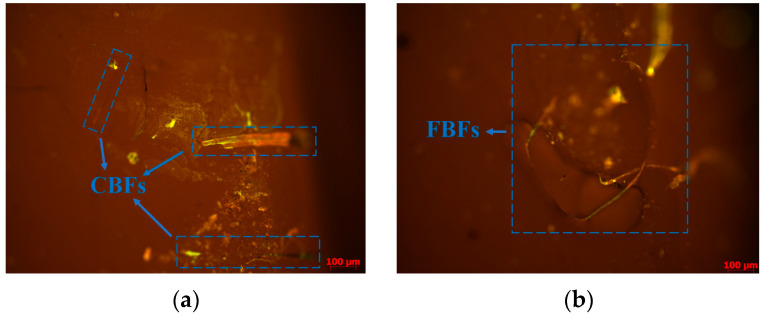
Fluorescent microscopic images: (**a**) CBFs asphalt binder; (**b**) FBFs asphalt binder.

At the microscopic scale, fluorescence microscopy can help to reveal the actual state of existence of fibers in asphalt binders to some extent. It can be seen from Figure 7 that both types of fibers have good dispersibility in asphalt binder and that no clumping phenomenon occurred. The CBFs still maintain their straight status, which could form a good bridging effect in the mixture. Nevertheless, the FBFs are cotton-like fibers formed by the curved basalt fiber monofilaments, which can help to form a three-dimensional network structure. Under this situation, the asphalt is adsorbed on the surface of the flocculent fiber, and the interface between the asphalt and aggregates is more stable, thus increasing the thickness of the asphalt film and the bond strength between asphalt and aggregates. At the same time, the microscopic picture of the fiber–asphalt binder could verify the view that there are clear boundary areas between fibers and asphalt, which can effectively increase the contact surface between fibers and the asphalt. Then, it can produce an anchoring effect when the fiber and asphalt interact so as to further improve the adhesion of fibers and asphalt.

#### 3.1.2. The Temperature Sweep Test

**Figure 8:**
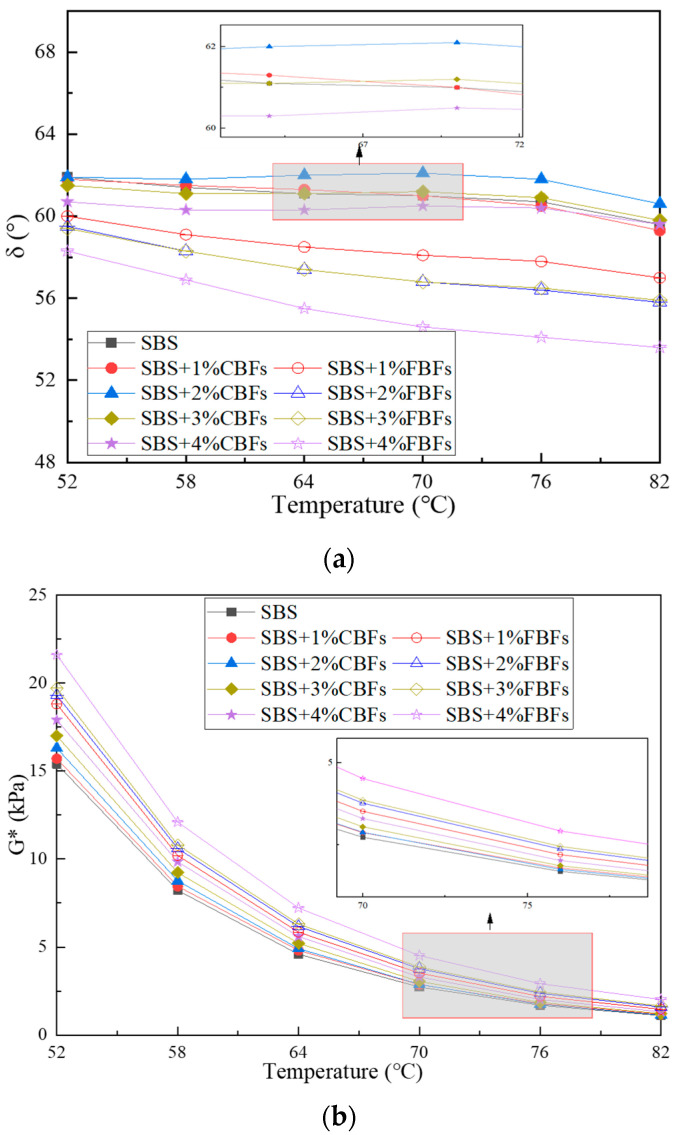

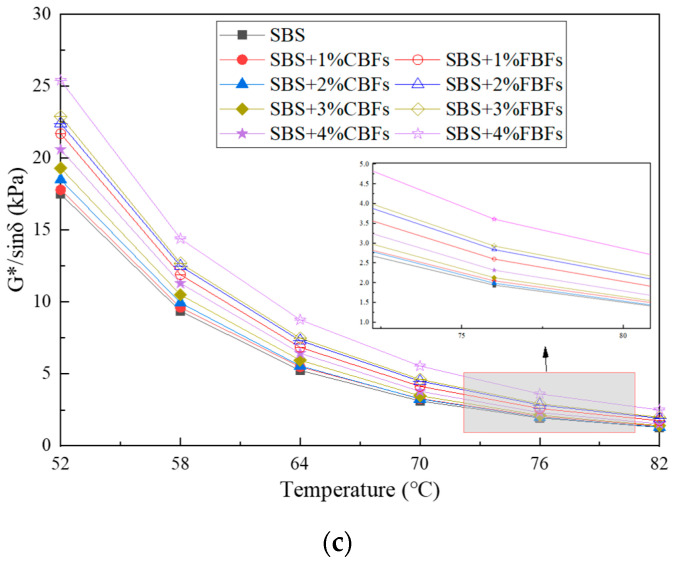
The results of the temperature sweep test: (**a**) δ; (**b**) G*; (**c**) G*/sinδ.

Comparative analyses of the δ, G* and G*/sinδ values of the three types of asphalt binders are depicted in Figure 8. When the temperature increases, the δ, G* and G*/sinδ values decrease.

After the addition of fiber, the phase angle of asphalt binder changes significantly but also retains the curve characteristics of asphalt binder. As to CBFs, when the fiber dosage is 1%, 2% and 3%, the phase angle curve at high temperature is not obviously different from that of SBS original asphalt, while at 4%, the phase angle curve has a dramatic decline. A possible reason for this phenomenon may be that enough CBFs can form a three-dimensional grid structure in the asphalt binder, which enhances the elasticity of the asphalt material and limits its fluidity under high-temperature conditions. For FBFs, the phase angle of asphalt binder under the condition of low dosage is also significantly lower than that of SBS original modified asphalt, and the phase angle decreases more significantly with the increase in fiber content. By looking back at Figure 1 and Figure 7, and comparing the appearance technical indicators of the two basalt fibers, it is clear that the specific surface area and gross volume of the flocculent basalt fibers with the same mass are larger. The flocculent basalt fiber with the same dosage makes it easier to stabilize the asphalt binders.

The results of complex shear modulus and rutting factor before and after adding basalt fibers are illustrated in Figure 8b,c. The results of G* and G*/sinδ values of asphalt binders decrease sharply with the increase in temperature, indicating that temperature is an important factor affecting the modulus values of asphalt binders, which also verifies the previous findings [23,25,40]. The curves between temperature and the complex modulus/rutting factor of fiber–asphalt binders are higher than those of SBS original asphalt, which indicates that fiber–asphalt binders have better deformation resistance at high temperatures. The curves of asphalt binders containing flocculent basalt fibers remain at the top all the time, and the index of asphalt binders with 4% flocculent basalt fiber increases remarkably, mainly because the high flexibility of flocculent basalt fiber can reduce the overall stiffness of asphalt binder.

Overall, it is obvious that the basalt fibers can minimize the influence of high temperature on the rheological properties of asphalt binders, and FBFs reveal more excellent ability in this respect, showing their better temperature sensitivity.

#### 3.1.3. MSCR Test

Previous studies [13,23,41] have shown that Jnr is used to reflect the nonlinear rheological response of asphalt under large stress, and it has a good correlation with the rutting resistance of asphalt mixture. When the stress increases, the R values of asphalt binders decrease hastily and the Jnr values increase drastically to a different degree, which indicates that the effect of “heavy load” on the resistance to permanent deformation of asphalt binders bears a resemblance to the “high temperature”. In other words, the stress and temperature have some kind of equivalence. Under the shear stress of 0.1 kPa at 64 °C, the rheological properties of asphalt binders are basically in the linear range. With the increase in stress level, the differences in R and Jnr between fiber–asphalt binders and the control group become more obvious. Moreover, the rheological properties of asphalt binders are likely to have entered into the nonlinear range, which should be able to simulate the situation of asphalt pavement under large loads. More specifically, the elastic recovery rates of asphalt binders are improved and non-recoverable creep compliances decrease after adding fibers. The improvement amplitude tends to be stable with the increase in the dosage. The R/Jnr curves of the chopped basalt fiber asphalt binder tend to be flat when the fiber dosage is 3%, whereas it tends to flatten out with a 2% fiber dosage for flocculent basalt fiber.

**Figure 9:**
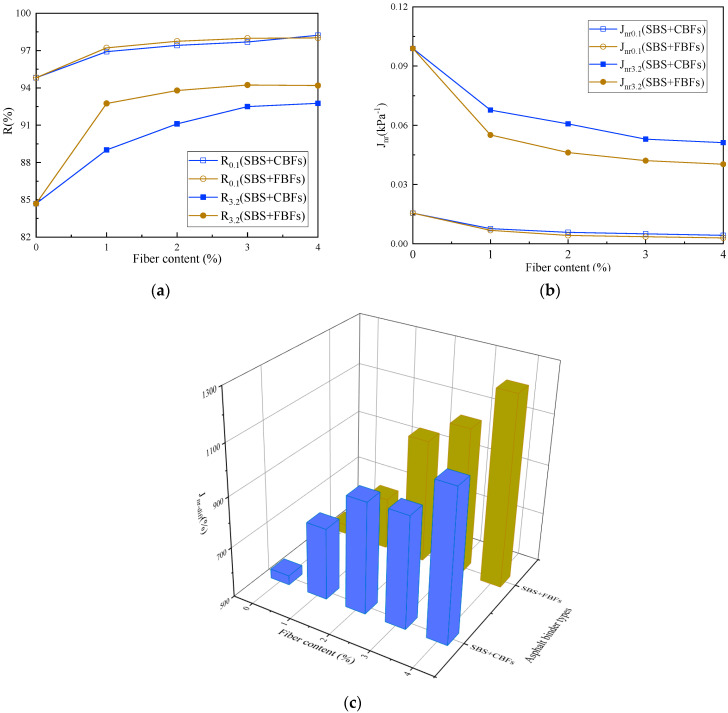
The MSCR results of different asphalt binders: (**a**) elastic recovery rate; (**b**) nonrecoverable creep compliance; (**c**) relative difference in nonrecoverable creep compliance.

For different fiber properties, under 3.2 kPa load, the elastic recovery rate of the FBFs asphalt binder is always slightly higher than that of the CBF asphalt binder. And the nonrecoverable creep compliance is inferior to that of the CBF asphalt binder, showing excellent high-temperature deformation resistance. By observing the stress-sensitive index Jnr-diff in Figure 9c, it is not difficult to find that the Jnr-diff values increase by 46.1%, 75.2%, 75.7% and 104.8%, respectively, with the increase in the dosage of CBFs. Meanwhile, as for FBFs, the Jnr-diff values increase by 31.6%, 84.4%, 101.8% and 133.6%, respectively. This yields increasingly good results on the data. The stress sensitivity of asphalt binder shows an increasing trend with the increase in fiber dosage, indicating that fiber is conducive to improving the stress sensitivity of asphalt binder. Meanwhile, superior results are seen for FBF asphalt binders, which is broadly in line with the results of the temperature sweep test.

### 3.2. Properties Evaluation of Fiber Reinforced Asphalt Mixtures

#### 3.2.1. Wheel Tracking Test

**Figure 10:**
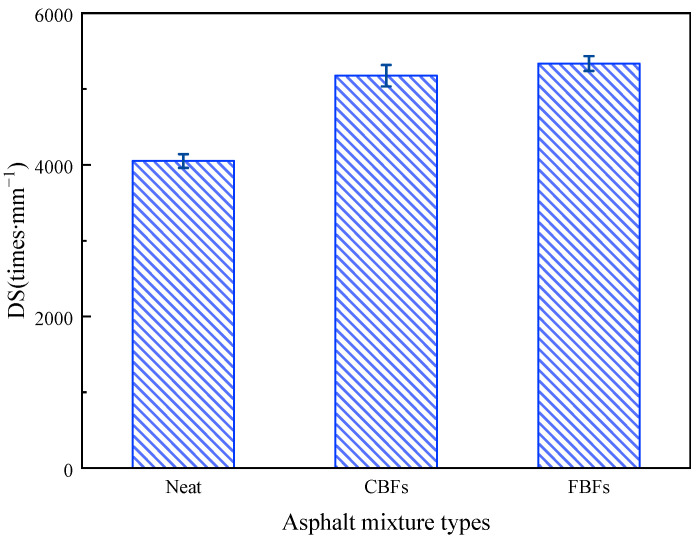
The dynamic stability results from the wheel tracking test of different samples.

The DS values of different asphalt mixtures are shown in Figure 10. It was obvious that the DS values increased after adding basalt fibers. Compared with the control group, the DS value of asphalt mixture with CBF increases from 4050 to 5175 times·mm^−1^, and the increasing extent is 28%. The DS value of asphalt mixture with FBFs increases from 4050 to 5335 times·mm^−1^, with a 32% increasing extent. Some scholars believe that fibers can better bond the asphalt and aggregate together, which could help to better resist the rolling loads [17,42,43]. Additionally, the highest DS values belong to the samples with FBFs, which show a slight advantage on the high temperature rutting resistance. This is because CBFs are dense and smooth, resulting in low fiber oil absorption rates. While the structure of the FBF is more complex, it can contribute to absorbing more asphalt. This phenomenon agrees with the results of the SEM test [23]. The high fiber oil absorption rate is conducive to preventing the segregation and overflow of the asphalt binder under high-temperature conditions.

#### 3.2.2. Uniaxial Penetration Test

**Figure 11:**
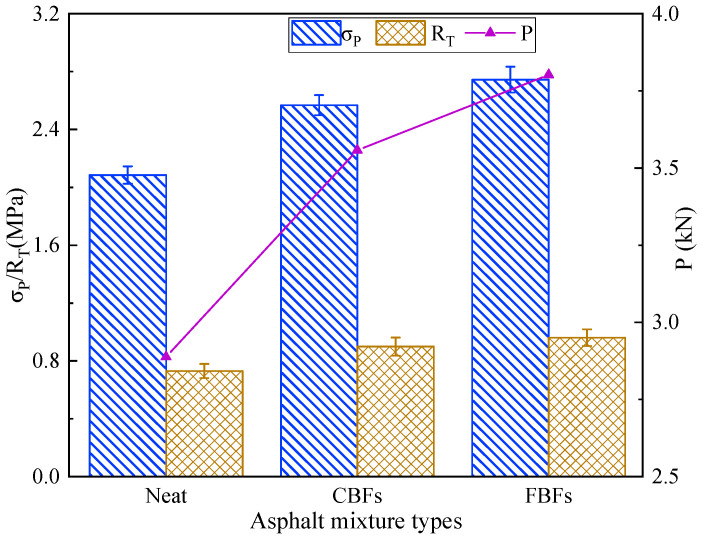
The uniaxial penetration test results of different samples.

Figure 11 illustrates the effects of the two basalt fibers on the uniaxial penetration test of the examined asphalt mixture samples. Compared with the control group, the P values of asphalt mixtures increase to 3.558 kN for mixtures containing CBFs and increase to 3.802 kN for mixtures containing FBFs. Simultaneously, the penetration strength value of the mixture increases by 23.2% for the mixture containing CBFs, and it increases by 31.6% for mixtures containing FBFs. The reason is mainly because the basalt fibers can absorb free asphalt in the mixture, which can be transformed into structural asphalt. Then, the aggregates are bonded to form a frame support in the mixture, thereby enhancing the shear deformation resistance of asphalt pavements.

Since the oil absorption rate of the FBFs is higher than that of the CBFs, the FBFs can overlap with each other and form a network structure in the mixture. This indicates that FBFs can better transfer the load and further reduce the structural damage caused by the stress concentration inside the asphalt pavements. This concurs well with the results of the wheel tracking test, which further proves the effect of basalt fibers on the high-temperature performance of the mixture and the effect of fiber morphologies on the mixtures.

#### 3.2.3. IDEAL-CT Test

**Figure 12:**
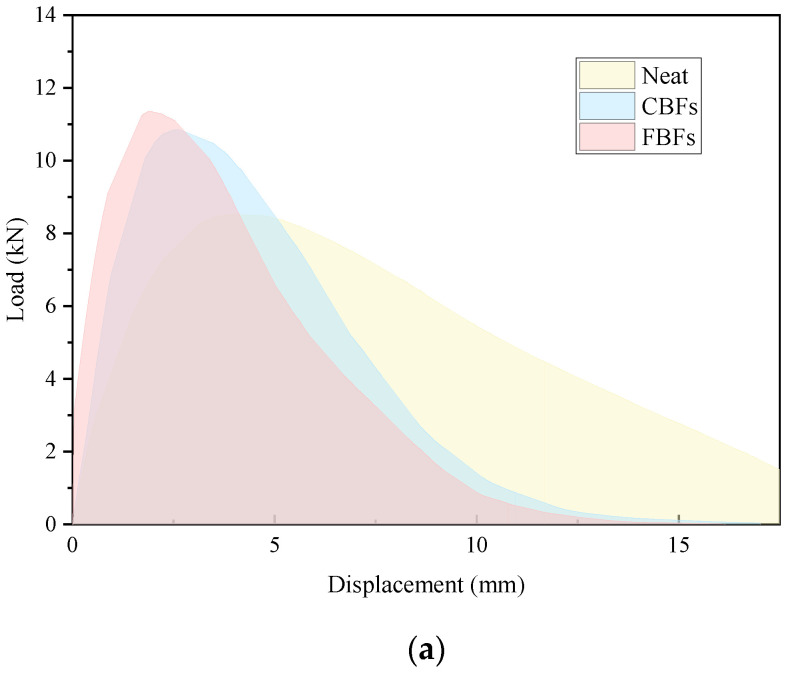

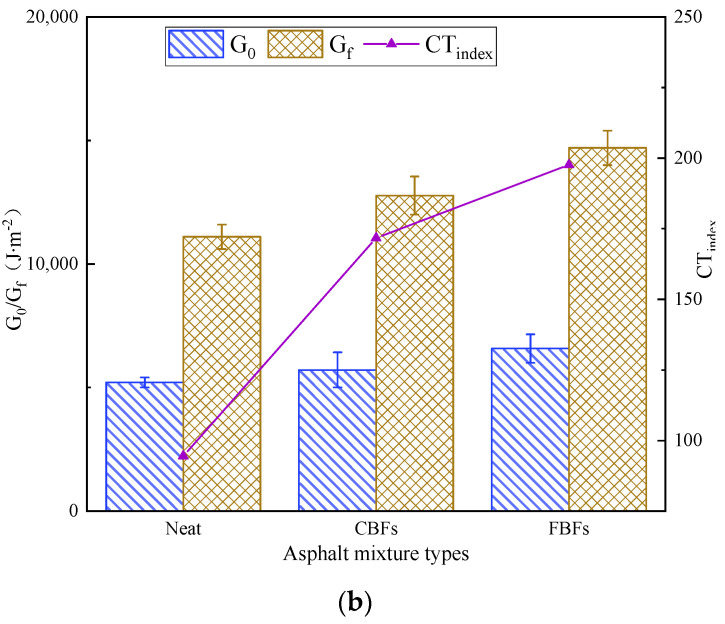
The IDEAL-CT results of different samples: (**a**) displacement–load curves; (**b**) initiation energy (G_0_), fracture energy (Gf) and CT_index_.

As is seen in Figure 12a, the displacement increases with the increase in the compressive load. Under the same load (before 100% peak load), the displacement of the samples with fibers is smaller, and it is more significant in the mixes with FBFs. In the whole process of the test, the initiation energy (G0), fracture energy and CT_index_ of the asphalt mixtures containing fibers are greatly improved when compared with the ordinary non-fiber asphalt mixture. After adding CBFs and FBFs, the initiation energy values of asphalt mixtures increase by 9.78% and 26.4%, the fracture energy values of mixtures increase by 15.1% and 32.5%, and the cracking index CT_index_ is elevated by 81.7% and 109.1%, respectively. Therefore, there is an effective enhancing effect on the initial cracking resistance of asphalt mixtures. This can be largely explained by the reason that basalt fiber is a strong inorganic material with high modulus, and the fibers can form an interconnected three-dimensional network structure. In the process of crack formation, the fibers help to transfer the internal stress of the mixture and increase the stiffness of the suspended dense skeleton structure. Moreover, the combination of basalt fibers and asphalt helps to delay the propagation speed of cracks [13,19,44].

In addition, from the perspective of composite materials, when additives with specific strength are incorporated, the mechanical strength of the composite will increase accordingly. Compared with the relevant test results of the asphalt mixture with fibers, the initiation energy, fracture energy and CT_index_ of the asphalt mixture with FBFs are all bigger than those of CBFs, which means that the crack growth rate of FBF-reinforced asphalt mixture is slower than that of CBFs reinforced asphalt mixture. This is because FBFs can help to absorb excess asphalt inside the asphalt mixture and can make the structural asphalt on the aggregates surface more tightly connected to each other. Since FBFs are randomly distributed in the asphalt mixture, it is helpful to connect fibers with the structural asphalt on the aggregate surface. Therefore, the cracking resistance of the asphalt mixture is better.

#### 3.2.4. Low-Temperature Bending Test

**Figure 13:**
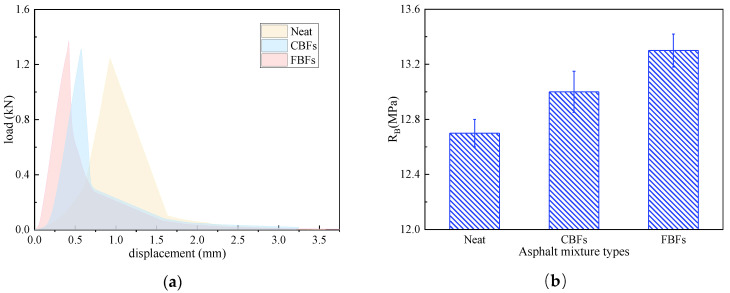

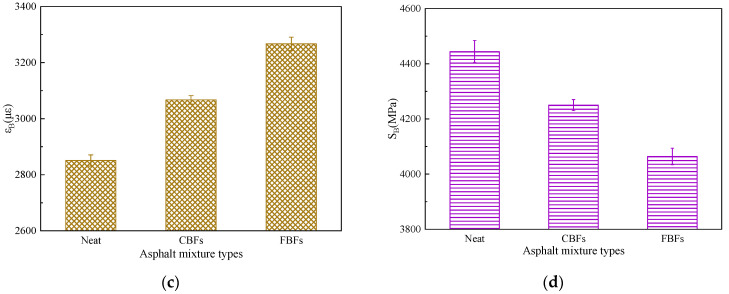
The low-temperature trabecular bending test results of different asphalt mixtures: (**a**) displacement–load curves; (**b**) flexural tensile strength; (**c**) failure strain; (**d**) bending stiffness modulus.

As is shown in Figure 13a, at −10 °C, the samples with FBFs have the maximum damage load, while the samples with CBFs have the minimal mid-span deflection. On the contrary, the control group has minimal damage load and maximum mid-span deflection. This demonstrates that the basalt-fiber-modified asphalt mixtures have stronger toughness and better crack resistance at low temperatures. The explanation may be that the asphalt becomes harder and more brittle under low-temperature conditions, which makes the adhesion between the fiber and the asphalt worse, resulting in the insignificant bridging effect. As shown in Figure 13b–d, after adding CBFs or FBFs, the flexural tensile strength (RB) values of asphalt mixtures increase by 2.36% and 4.72%, the failure strain (εB) values enlarge to 3067 με and 3267 με, and the bending stiffness modulus (SB) of asphalt mixture reduces by 4.37% and 8.55%, respectively. As mentioned before, the oil absorption rate of FBFs is relatively high, which promotes the flexibility of the asphalt mixture and forms more asphalt films. As a result, FBFs play a better role in filling and healing the micro-cracks between the aggregates.

#### 3.2.5. Water Immersion Stability Test and Freeze–Thaw Splitting Test

**Figure 14:**
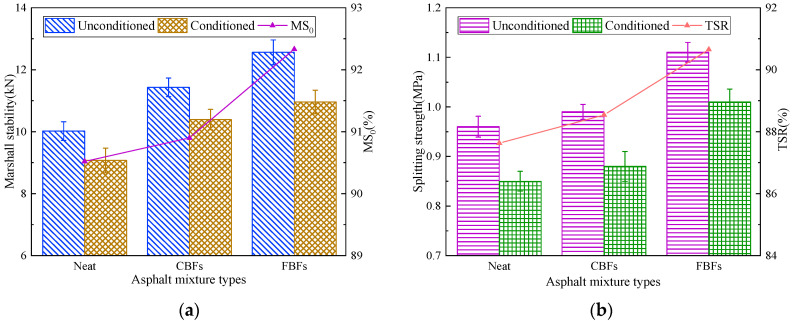
Moisture susceptibility test results: (**a**) water immersion stability test; (**b**) freeze–thaw splitting test.

The shear force transferred from the road surface under traffic load accelerates the stripping and spalling of aggregates, leading to the aggravation of water damage [45]. Water immersion stability test results have been presented in Figure 14a. Compared with the control group, the Marshall stability values of the asphalt mixture with CBFs before and after the immersion process are slightly increased and the MS0 values are almost unchanged. However, the relevant indexes of the asphalt mixture after the adding FBFs are improved evidently, and the MS0 value increases by about 20.0%. The explanation may be that the presence of fibers plays a crucial role in increasing the consistency of the asphalt binder and improving the binding effect with aggregates. As mentioned before, FBFs can better absorb the light components in asphalt, increasing the thickness of asphalt film. In addition. It could then improve the bond strength as well as prevent water from entering the interface between asphalt and aggregates effectively.

According to the results of freeze–thaw splitting test (Figure 14b), the TSR value of the asphalt mixture with CBFs is 88.54%, which increases by 1.04% more than that of the non-fiber asphalt mixture. Conversely, the TRS value of the mixture with FBFs reaches 90.65%, which is about 3% higher than that of the control group. It is evident that the TSR value of the asphalt mixture with FBFs increases more obviously than that with CBFs, which shows the same pattern as the result of the immersion Marshall test. On the one hand, the oil absorption rate of FBFs is higher than that of CBF, and the higher OAC can make the water less likely to be immersed in the asphalt mixture. On the other hand, FBFs maintain high strength characteristics and show a better reinforcement effect than the ordinary dense graded asphalt mixture. In a word, FBFs can help to control the spread of asphalt pavement cracks and reduce the occurrence of water damage by absorbing part of the fracture energy during the service of asphalt pavement.

### 3.3. Comprehensive Analysis

**Figure 15:**
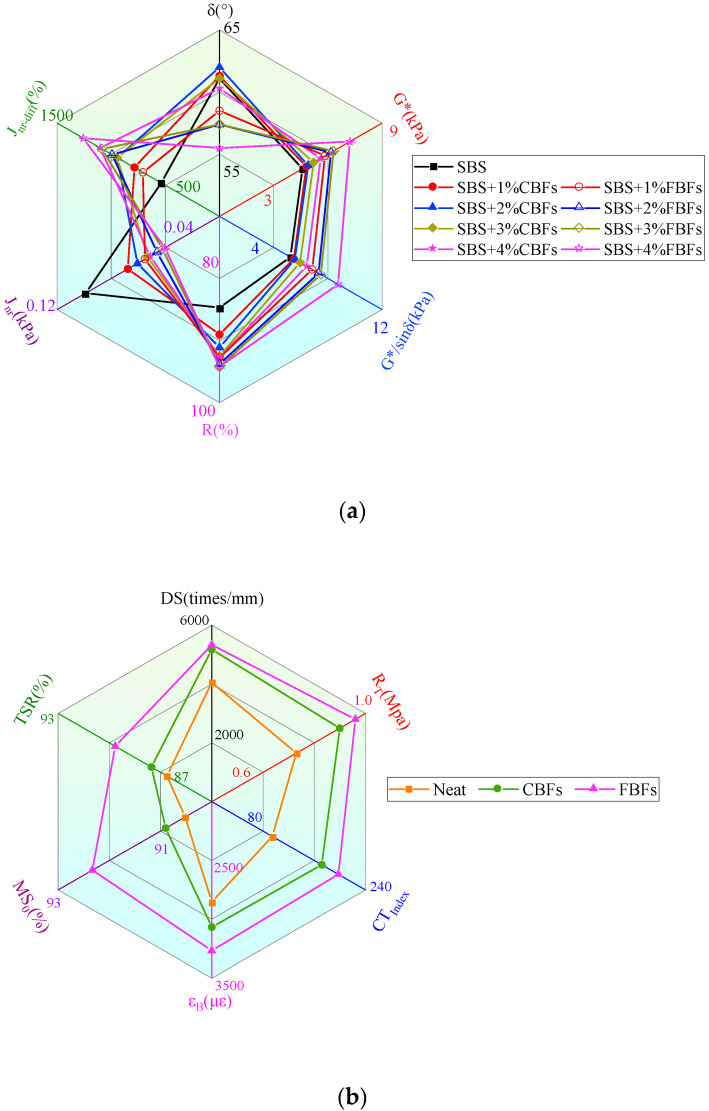
Radar maps: (**a**) rheological behavior of different asphalt binders with 1% fiber dosage; (**b**) pavement performance of asphalt mixtures.

The rheological properties of different asphalt binders and road performance of asphalt mixtures are multidimensionally expressed by radar maps, as shown in Figure 15. In terms of asphalt binders, δ, G*, G*/sinδ (at 64 °C), and R, Jnr, Jnr-diff (under 3.2 kPa at 64 °C) were chosen to represent the rheological properties. For the asphalt mixtures, DS and R_T_ are considered to represent the high-temperature stabilities, CT_index_/ε_B_ is selected to stand for the crack resistance at medium/low temperature, and MS0 as well as TSR are considered to represent the moisture susceptibilities.

The improvement effects of different basalt fiber morphologies on the asphalt binders or asphalt mixtures are various. In Figure 15a, the rheological properties of asphalt binders mixed with CBFs are slightly higher than that of ordinary SBS-modified asphalt, while the elasticity of asphalt binders mixed with FBFs is distinctly increased, and the deformation resistance at high temperatures is significantly improved. As seen from the radar chart of asphalt mixture pavement performance (Figure 15b), FBFs have a more favorable influence on the comprehensive performance of asphalt mixture, which could prolong the service life of asphalt pavement. However, the impact of CBFs is slightly inferior to that of FBFs. Therefore, in addition to the traditional parameters such as fiber dosage, length, diameter, etc., the morphology of the fibers also has a crucial effect on the properties of asphalt binders or asphalt mixtures.

**Figure 16:**
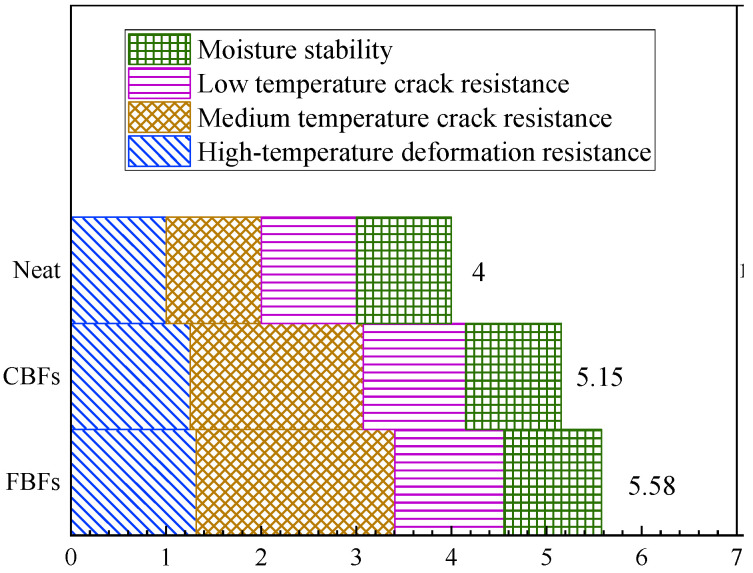
The sum results of normalized values.

In order to evaluate the effect of different morphologies of basalt fiber on the properties of asphalt mixtures more directly, the normalization method was used for quantitative analysis. The performance index of the mixture without basalt fiber is used as the reference material, which was marked as “1”. Then, the increase or decrease in the properties of the other samples relative to the baseline was obtained, where the increase was positive and the decrease was negative, respectively. The selection of asphalt mixture indicators is similar to that of radar maps. It should be noted in particular that the average changes in DS and RT are considered as the changes in the high-temperature deformation resistance, and the average changes in MS_0_ and TSR are considered as the changes in moisture stability. Finally, the sum of normalized values of mixtures was calculated and depicted in Figure 16.

The results verify that the two different morphologies of basalt fibers can significantly improve the overall performance of the asphalt mixture. The normalized analysis values of high-temperature deformation resistance and medium-temperature crack resistance are between 1.255 and 1.316 and 1.817 and 2.091, respectively. This indicates that basalt fiber can greatly enhance the medium- and high-temperature performance of asphalt mixture. Meanwhile, the normalized values of the low-temperature crack resistance and moisture susceptibility range from 1.076 to 1.146 and 1.007 to 1.027, respectively. This phenomenon suggests that basalt fiber can improve the corresponding properties of asphalt mixtures, although the amplitude is not obvious. Simultaneously, in terms of the normalized sum results, mixtures with FBFs show better enhancement capability. Consequently, morphology should also be considered when selecting fibers in engineering practice.

## 4. Conclusions

In this paper, two basalt fibers (CBFs and FBFs) were blended with SBS-modified asphalt to prepare the fiber-reinforced asphalt binder. The microscopic morphology and the rheological properties of fiber–asphalt binders were studied by a fluorescence microscopy test, a temperature sweep test and an MSCR test. Subsequently, two kinds of fibers were mixed into asphalt mixtures by the dry method, and the mixture samples were tested to investigate the pavement performance. The main conclusions can be summarized as follows:

(1) Adding basalt fibers with different morphologies makes the volume parameters of the asphalt mixture change to some extent. With the same amount of fiber in asphalt mixtures, FBFs can absorb more structural asphalt than CBFs, and the OAC values and Marshall stability values of asphalt mixture with FBFs are slightly higher than those with CBFs.
(2) Both basalt fibers can be well dispersed in the asphalt binder and form a three-dimensional grid structure. Compared with CBFs, the larger specific surface area of FBFs with the same weight content makes the asphalt binder structure more stable. And the additives of fibers could increase the elasticity, decrease the viscosity and improve the temperature sensitivity of asphalt materials. An asphalt binder containing FBFs shows better high-temperature performance and elastic recovery ability, followed by CBFs.
(3) The enhancement effect of FBFs on the medium/high-temperature properties and moisture susceptibility of the asphalt mixture is obviously better than that of CBFs. The small volume of air void in the FBF asphalt mixture also ensures its resistance to water damage. However, FBFs only have a slight advantage in terms of low-temperature cracking resistance. Therefore, if the fiber is used in areas with high requirements for low-temperature crack resistance, the fibers with lower costs could be chosen.
(4) FBFs have a superior impact on the rheological properties of asphalt binders and the comprehensive pavement performance of asphalt mixtures. The morphology of the fiber should be taken into account, especially in the areas having middle or high temperatures.

## Figures and Tables

**Table 1:** Basic properties of different basalt fibers.

Fiber Types	Density (g/cm^−3^)	Length (mm)	Diameter (μm)	Tensile Strength (MPa)	Water Content (%)	Oil Absorption Rate (%)
CBFs	2.762	6	13–16	2831	<0.1	104
FBFs	2.817	4–7	6–8	2804	<0.1	230

**Table 2:** Components of asphalt mixtures and Marshall test results.

Fiber Stabilizers	Fiber Contents (%)	OAC (%)	VV (%)	VMA (%)	VFA (%)	Marshall Stability (kN)	Flow Values (cm)
Neat	/	4.9	3.73	13.84	72.26	10.02	3.1
CBFs	0.3	5.1	3.99	14.72	72.87	12.03	3.2
FBFs	0.3	5.2	3.87	14.61	73.52	12.56	3.3

## Data Availability

The original contributions presented in the study are included in the article, further inquiries can be directed to the corresponding author.

## References

[1] Kim J.S., Lim J.H., Huh Y. (2013). Melt-spinning Basalt Fibers Based on Dielectric Heating and Steady-state Process Characteristics. Fibers Polym..

[2] Nikitina F.M., Kogano V. (1994). Solubility of chrysotile asbestos and basalt fibres in relation to their fibrogenic and carcinogenic action. Environ. Health Perspect..

[3] Zhao G., Li M., Wang S., Peng X., Wang L., Li X., Zhao Y., Gao Y., Zhang Y., Zheng J. (2024). Effect of interlaminar basalt fiber veil reinforcement on mode I fracture toughness of basalt fiber composites. Polym. Compos..

[4] Yang C., Liu L., Liu Z., Huang Y., Yu S., Fu Y. (2023). Study on the mechanism of bond strength generation and debonding failure between basalt fiber and asphalt based on molecular dynamics. Case Stud. Constr. Mater..

[5] Abiola O.S., Kupolati W.K., Sadiku E.R., Ndambuki J.M. (2014). Utilisation of natural fibre as modifier in bituminous mixes: A review. Constr. Build. Mater..

[6] Sarayu K., Gopinath S., Ramachandra M.A., Iyer N.R. (2016). Structural stability of basalt fibers with varying biochemical conditions-A invitro and invivo study. J. Build. Eng..

[7] Liu Y., Zhang M., Liu H., Tian L., Liu J., Fu C., Fu X. (2022). Properties of Basalt Fiber Core Rods and Their Application in Composite Cross Arms of a Power Distribution Network. Polymers.

[8] Li Y., Wang Q., Xu S., Song Q. (2023). Study of eco-friendly fabricated hydrophobic concrete containing basalt fiber with good durability. J. Build. Eng..

[9] Fiore V., Scalici T., Bella G.D., Valenza A. (2015). A review on basalt fibre and its composites. Compos. B Eng..

[10] Zhou S., Gao J., Wang J., Wang S., Liu Y. (2019). Polydopamine-coupling of carbon nanotubes onto microscaled basalt fiber to enhancing mechanical, thermal and tribological properties of composite materials. Mater. Res. Express.

[11] Celauro C., Praticò F.G. (2018). Asphalt mixtures modified with basalt fibres for surface courses. Constr. Build. Mater..

[12] Xie T., Wang L. (2023). Optimize the design by evaluating the performance of asphalt mastic reinforced with different basalt fiber lengths and contents. Constr. Build. Mater..

[13] Pei Z., Lou K., Kong H., Wu B., Wu X., Xiao P., Qi Y. (2021). Effects of Fiber Diameter on Crack Resistance of Asphalt Mixtures Reinforced by Basalt Fibers Based on Digital Image Correlation Technology. Materials.

[14] Lou K., Xiao P., Wu B., Kang A., Wu X., Shen Q. (2021). Effects of fiber length and content on the performance of ultra-thin wearing course modified by basalt fibers. Constr. Build. Mater..

[15] Sun X., Qin X., Chen Q., Ma Q. (2018). Investigation of enhancing effect and mechanism of basalt fiber on toughness of asphalt material. Pet. Sci. Technol..

[16] Zhang J., Huang W., Zhang Y., Lv Q., Yan C. (2020). Evaluating four typical fibers used for OGFC mixture modification regarding drainage, raveling, rutting and fatigue resistance. Constr. Build. Mater..

[17] Wang W., Cheng Y., Tan G. (2018). Design Optimization of SBS-Modified Asphalt Mixture Reinforced with Eco-Friendly Basalt Fiber Based on Response Surface Methodology. Materials.

[18] Gao L., Hu G., Xu N., Fu J., Xiang C., Yang C. (2015). Experimental Study on Unconfined Compressive Strength of Basalt Fiber Reinforced Clay Soil. Adv. Mater. Sci. Eng..

[19] Lou K., Wu X., Xiao P., Kang A., Wu Z., Xia Y. (2021). Comprehensive Study about Effect of Basalt Fiber, Gradation, Nominal Maximum Aggregate Size and Asphalt on the Anti-Cracking Ability of Asphalt Mixtures. Appl. Sci..

[20] Li Z., Shen A., Wang H., Guo Y., Wu H. (2020). Effect of basalt fiber on the low-temperature performance of an asphalt mixture in a heavily frozen area. Constr. Build. Mater..

[21] Cetin A., Evirgen B., Karslioglu A., Tuncan A. (2021). The Effect of Basalt Fiber on the Performance of Stone Mastic Asphalt. Period. Polytech-Civ..

[22] Thanh D.V., Feng C.P. (2013). Study on Marshall and Rutting test of SMA at abnormally high temperature. Constr. Build. Mater..

[23] Wu B., Pei Z., Luo C., Xia J., Chen C., Kang A. (2022). Effect of different basalt fibers on the rheological behavior of asphalt mastic. Constr. Build. Mater..

[24] Shi C., Wang J., Sun S., Lv D., Xu L., Zhang S. (2023). Research on properties of basalt fiber-reinforced asphalt mastic. Front. Mater..

[25] Kou C., Chen Z., Kang A., Zhang M., Wang R. (2022). Rheological behaviors of asphalt binders reinforced by various fibers. Constr. Build. Mater..

[26] Gu Q., Kang A., Li B., Xiao P., Ding H. (2022). Effect of fiber characteristic parameters on the high and low temperature rheological properties of basalt fiber modified asphalt mortar. Case Stud. Constr. Mater..

[27] (2011). Standard Test Methods of Bitumen and Bituminous Mixtures for Highway Engineering.

[28] (2011). Test Methods of Aggregate for Highway Engineering.

[29] Liu K., Li T., Wu C., Jiang K., Shi X. (2021). Bamboo fiber has engineering properties and performance suitable as reinforcement for asphalt mixture. Constr. Build. Mater..

[30] Lou K., Xiao P., Ong G.P., Li B., Kang A., Wu Z. (2024). Micromechanical behavior of single fiber-asphalt mastic interface: Experimental studies by self-designed innovative pullout test. Constr. Build. Mater..

[31] Wang S., Kang A., Xiao P., Li B., Fu W. (2019). Investigating the Effects of Chopped Basalt Fiber on the Performance of Porous Asphalt Mixture. Adv. Mater. Sci. Eng..

[32] (2020). Standard Method of Test for Determining the Rheological Properties of Asphalt Binder Using a Dynamic Shear Rheometer (DSR).

[33] (2020). Standard Method of Test for Multiple Stress Creep Recovery (MSCR) Test of Asphalt Binder Using a Dynamic Shear Rheometer (DSR).

[34] Peng Y., Xia S., Xu Y., Lu X., Li Y. (2021). Discrete-Element Modeling of Influence of Void Characteristics on Uniaxial Penetration Strength of Asphalt Mixtures. J. Mater. Civ. Eng..

[35] (2017). Specifications for Design of Highway Asphalt Pavement.

[36] Ma Z., Liu L., Sun L. (2018). Investigation of top-down cracking performance of in-situ asphalt mixtures based on accelerated pavement testing and laboratory tests. Constr. Build. Mater..

[37] (2019). Standard Test Method for Determination of Cracking Tolerance Index of Asphalt Mixture Using the Indirect Tensile Cracking Test at Intermediate Temperature.

[38] Chen H., Zhang Y., Bahia H.U. (2021). The role of binders in mixture cracking resistance measured by ideal-CT test. Int. J. Fatigue.

[39] Zhang K., Li W., Han F. (2019). Performance deterioration mechanism and improvement techniques of asphalt mixture in salty and humid environment. Constr. Build. Mater..

[40] Gao L., Wang Z., Liu Y., Zheng J., Li H. (2019). Influence of Binder Property and Mortar Thickness on High-Temperature Performance of Cold Recycled Mixtures with Asphalt Emulsion. Materials.

[41] Li S., Shi X., Si C., Bao B., Hu M. (2023). Correlation between the Rheological Properties of Asphalt Mortar and the High-Temperature Performance of Asphalt Mixture. Coatings.

[42] Pirmohammad S., Amani B., Shokorlou Y.M. (2020). The effect of basalt fibres on fracture toughness of asphalt mixture. Fatigue. Fract. Eng. Mater. Struct..

[43] Chen H., Xu Q., Chen S., Zhang Z. (2009). Evaluation and design of fiber-reinforced asphalt mixtures. Mater. Des..

[44] Yang K., He Z., Li D., Xu H., Kong L. (2021). Experimental Study on Basalt Fiber Crack Resistance of Asphalt Concrete Based on Acoustic Emission. Materials.

[45] Wang W., Wang L., Xiong H., Luo R. (2019). A review and perspective for research on moisture damage in asphalt pavement induced by dynamic pore water pressure. Constr. Build. Mater..

